# Incidence of pancreatitis, secondary causes, and treatment of patients referred to a specialty lipid clinic with severe hypertriglyceridemia: a retrospective cohort study

**DOI:** 10.1186/1476-511X-10-157

**Published:** 2011-09-11

**Authors:** Supna Sandhu, Ahmad Al-Sarraf, Catalin Taraboanta, Jiri Frohlich, Gordon A Francis

**Affiliations:** 1 Department of Medicine, Healthy Heart Program Prevention Clinic, UBC James Hogg Research Centre, Providence Heart + Lung Institute, St. Paul's Hospital, University of British Columbia, 1081 Burrard St., Vancouver, BC V6Z 1Y6 Canada; 2 Department of Pathology and Laboratory Medicine, Healthy Heart Program Prevention Clinic, UBC James Hogg Research Centre, Providence Heart + Lung Institute, St. Paul's Hospital, University of British Columbia, 1081 Burrard St., Vancouver, BC V6Z 1Y6 Canada

**Keywords:** Hypertriglyceridemia, triglycerides, pancreatitis, dysglycemia, diabetes, fibrates

## Abstract

**Background:**

Severe hypertriglyceridemia (HTG) is one cause of acute pancreatitis, yet the level of plasma triglycerides likely to be responsible for inducing pancreatitis has not been clearly defined.

**Methods and Results:**

A retrospective cohort study was conducted on patients presenting non-acutely to the Healthy Heart Program Lipid Clinic at St. Paul's Hospital with a TG level > 20 mM (1772 mg/dl) between 1986 and 2007. Ninety-five patients with TG > 20 mM at the time of referral were identified, in who follow up data was available for 84. Fifteen patients (15.8%), with a mean outpatient TG level of 38.1 mM, had a history of acute pancreatitis. Among 91 additional patients with less severe HTG, none had a history of pancreatitis when TG were between 10 and 20 mM. Among patients with TG > 20 mM on presentation, 8 (8.5%), with a mean TG level of 67.8 mM, exhibited eruptive xanthomata. A diet high in carbohydrates and fats (79%) and obesity (47.6%) were the two most frequent secondary causes of HTG at initial visit. By 2009, among patients with follow up data 53% exhibited either pre-diabetes or overt Type 2 diabetes mellitus. Upon referral only 23 patients (24%) were receiving a fibrate as either monotherapy or part of combination lipid-lowering therapy. Following initial assessment by a lipid specialist this rose to 84%, and remained at 67% at the last follow up visit.

**Conclusions:**

These results suggest hypertriglyceridemia is unlikely to be the primary cause of acute pancreatitis unless TG levels are > 20 mM, that dysglycemia, a diet high in carbohydrates and fats, and obesity are the main secondary causes of HTG, and that fibrates are frequently overlooked as the drug of first choice for severe HTG.

## Background

Hypertriglyceridemia (HTG), classically defined as fasting plasma triacylglycerols (triglycerides, TG) > 2.3 mM or 200 mg/dl, or 1.7 mM (150 mg/dl) in the definition of metabolic syndrome [1], is a common laboratory finding. Severe hypertriglyceridemia, *e.g*., TG > 20 mM (1772 mg/dl), is much rarer and almost always caused by a combination of inherited and secondary factors [2,3]. Genetic disorders leading to hypertriglyceridemia include familial combined hyperlipidemia, familial hypertriglyceridemia, remnant removal disease (Type 3 dyslipidemia), deficiencies of lipoprotein lipase or apolipoprotein CII, and more recently characterized mutations including variants of apolipoprotein A5 [4,5]. The most common secondary contributors to severe hypertriglyceridemia include poorly controlled diabetes mellitus, obesity, high fat and simple carbohydrate diet, excess alcohol consumption, hypothyroidism, and medications including thiazide diuretics, β-blockers, oral estrogen, retinoids, and anti-retroviral agents [2,3].

Patients with severe HTG may present with classic findings such as abdominal pain or overt pancreatitis, eruptive or palmar xanthomas, lipemia retinalis, or they may be asymptomatic [2,3]. The most significant complication of severe HTG is acute pancreatitis, which may lead to pancreatic necrosis and death [2,6]. The incidence of classic signs and symptoms of HTG, including pancreatitis, however, has not been determined in patients presenting with severe HTG. In addition, the level of plasma triglycerides at which acute pancreatitis can be ascribed specifically to the presence of HTG has not been reported.

The purpose of this study was to determine the frequency of physical signs and symptoms of HTG including pancreatitis among patients with severe HTG referred to a specialty lipid disorders clinic over a 21-year period. Specifically, our study attempted to determine: (a) the frequency of classical signs and symptoms associated with severe HTG; (b) the most common secondary factors contributing to TG > 20 mM (1772 mg/dl); (c) differences in treatment for severe HTG between referring physicians and lipid clinic specialists; and (d) changes in the lipid profile of patients with severe HTG followed at a specialty lipid clinic. Overall, we found an absence of pancreatitis unless TG were > 20 mM, a relatively low incidence of classic clinical findings of HTG such as eruptive xanthomas, the presence of diabetes or pre-diabetes in the majority of HTG subjects, and a tendency of non-lipid specialists to overutilize statins and underutilize fibrates as their first-line treatment for severe HTG.

## Results

### Patient Demographics (Table 1)

Between 1986 to 2007 the clinic saw a total of 95 patients with TG ≥ 20 mM at the time of first referral. The mean age ± standard deviation was 54.2 ± 11.9 years, with 70 patients (73.7%) being male and 75 patients (78.9%) Caucasian.

**Table 1:** Demographic Information for HTG Patients At Initial Clinic Visit

		n/N	%
Mean Age (yrs)	54.2 +/- 11.9	95/95	
Gender	Male	70/95	73.7
	Female	25/95	26.3
Ethnicity	Caucasian	75/95	78.9
	South Asian	12/95	12.6
	Chinese	4/95	4.2
	Other	4/95	4.2

### History of Pancreatitis

Fifteen patients (15.8%) had a history of pancreatitis prior to referral to the clinic. Of these, the mean TG level at the time of non-acute presentation to the clinic was 38.13 mM [median 30.91 mM (IQ 25.6 - 52.2)], with the lowest referral TG level associated with prior pancreatitis being 20.5 mM (1815 mg/dl). Peak TG levels at the time of acute pancreatitis were not available for this analysis. Analysis of an additional cohort of 91 patients with TG levels between 10 and 20 mM (886 - 1771 mg/dl) at time of presentation to clinic revealed a history of pancreatitis in only 3 patients. In these 3, levels of TG at the time of acute pancreatitis were available, and all were > 20 mM (1771 mg/dl). As such, we conclude that pancreatitis is unlikely to occur as a result of hypertriglyceridemia unless TG are > 20 mM acutely.

### Prevalence of dysglycemia (Table 2)

Thirty patients (31.6%) had a prior diagnosis of diabetes mellitus (DM). Of these, HbA1 c levels were not routinely available, however the majority (23/30) had poor control, with a mean FBS of 9.97 ± 4.37 mM (179 ± 79 mg/dL) at presentation to the clinic, and only 11 were on antihyperglycemic medication. An additional 5 patients (5.3%) were diagnosed with DM at their first clinic visit. Four new diagnoses of DM were made during the follow up period. In the entire group with severe hyperTG, 39 patients (41.0%) had a diagnosis of Type 2 DM by 2009. An additional 11 patients (11.6%) had impaired fasting glucose, giving a total of 50 patients (52.6%) having impaired glucose metabolism. At the latest visit, 82% of patients with a diagnosis of DM were taking antihyperglycemic medication.

**Table 2:** Frequency of Diabetes and Elevated Fasting Blood Sugar (FBS) in HTG Clinic Patients

		n/N	%
At Initial Visit:	Past DM Diagnosis	30/95	31.6
	FBS > 7 mM	23/30	76.7
	New DM Diagnosis	5/52	9.6
At Interim:	New DM Diagnosis	4/60	6.8
At Latest Visit:	Total DM	39/95	41.1
	Impaired Fasting Glucose	11/95	11.6
	Total with Dysglycemia	50/95	52.6

### Other risk factors for hypertriglyceridemia and personal/family history of CVD (Table 3)

Dietary assessment by clinic dietitians was performed using three day food records plus a nutrient frequency questionnaire. Fat intake was considered high if >35% and carbohydrate intake high if >55% of total calories [7]. Seventy-five patients (78.9%) had a diet high in fat and carbohydrates based on these criteria. Forty-two patients (49.4%) had no regular exercise. Alcohol consumption of >14 drinks/week or 7 - 14 drinks/week were recorded in 11 (11.6%) and 8 (8.4%) patients, respectively. Of the 15 (17.1%) patients previously diagnosed with hypothyroidism, 5 were inadequately controlled based on a TSH > 5 uIU/mL at presentation. No new diagnoses of hypothyroidism were made during follow up of this cohort. Twenty-four patients (25.3%) had smoked cigarettes within the past year. Sixteen patients (16.9%) were on beta-blockers, 6 (6.4%) were on estrogen therapy, 4 (4.3%) were on anti-retroviral therapy, and 3 (3.2%) were on a thiazide diuretic. Twelve patients (12.6%) had a personal history of coronary artery disease and 5 (5.3%) of peripheral vascular disease. Forty-seven patients (49.5%) indicated a history of premature vascular disease in first-degree relatives; only 15 (15.8%) were aware of other family members having dyslipidemia; however, this information was unknown to many patients.

**Table 3:** Frequency of Risk Factors in HTG Patients at Initial Clinic Visit

		n/N	%
High fat/sugar diet:		75/95	79
			
Physical Exercise:	>5/wk	13/85	15.3
	3-5/wk	15/85	17.6
	< 3/wk	15/85	17.6
	none	42/85	49.4
			
Alcohol Consumption:	>14 drinks/wk	11/95	11.6
	7-14 drinks/wk	8/95	8.4
	1-7 drinks/wk	33/95	34.7
	none	43/95	45.3
			
Diagnosed Hypothyroidism:		15/95	17.1
	with TSH>5	5/15	33.3
			
Currently Smoking:		24/95	25.3
			
Medications:	B-blocker	16/95	16.9
	Estrogen	6/95	6.4
	Anti-retrovirals	4/95	4.3
	Thiazide diuretic	3/95	3.2
			
Personal History:	Premature CAD	12/95	12.6
	PVD	5/95	5.3
			
Family History:	Premature CVD	47/95	49.5
	Dyslipidemia	15/95	15.8

CAD = Coronary Artery Disease; PVD = Peripheral Vascular Disease;

CVD = Cardiovascular Disease; TSH = Thyroid Stimulating Hormone

### Physical findings (Table 4)

Obesity, defined as a body-mass-index > 30 kg/m^2^, was present in 45 patients (47.4%), 57% of whom were previously diagnosed with diabetes. Overweight, as defined by a body-mass-index of 25-30 kg/m^2 ^was present in an additional 29.5%, with only 23% of the cohort having a BMI less than 25 kg/m^2^. Eight patients (8.5%) presented with eruptive xanthomas, occurring with a range of TG levels of 20.5 - 171.9 mM, and a mean TG level of 67.8 mM [median 51.2 mM (IQ 27.55 - 97.85)]. Of these 8 patients, 2 were also felt to have lipemia retinalis (the only 2 of the entire cohort in whom this was noted, occurring at TG levels of 25.6 and 54.3 mM), 5 were obese, and 4 were diabetic. Three patients (3.2%) had palmar xanthomas. Corneal arcus was observed in 24 patients (25.3%). Three patients had abdominal tenderness at the initial visit, one of whom had a previous history of pancreatitis, and 6 were noted to have hepatomegaly.

**Table 4:** Frequency of Clinical Findings in HTG Patients

		n/N	%
Body Mass Index:	> 30 kg/m2	45/95	47.4
	25-30 kg/m2	28/95	29.5
	< 25 kg/m2	22/95	23.2
Dermatological:	Eruptive Xanthomas	8/95	8.5
	Palmar Xanthomas	3/95	3.2
Ophthamalogical:	Corneal Arcus	24/95	25.3
	Lipemia Retinalis	2/95	2.1
Gastrointestinal:	Abdominal tenderness	3/95	3.2
	Hepatomegaly	6/95	6.3

### Pre-Clinic and In-Clinic Treatment (Table 5)

At the initial visit 23 patients (24.2%) were taking a fibrate, with 17 on fibrate monotherapy, 4 on fibrate-statin, 1 on fibrate-niacin, and 1 on fibrate-omega-3 fatty acids (fish oil). Sixteen patients (16.8%) were referred taking statin monotherapy. Fifty-two patients (54.7%) were taking no hypolipidemic therapy, either due to lack of initiation or a history of lipid therapy intolerance. All patients received extensive dietary counseling and encouraged to limit dietary fats and simple carbohydrates as well as alcohol consumption. After the initial visit 80 patients (84.2%) were taking fibrate therapy, with 54 (56.8%) on fibrate monotherapy, 16 (16.8%) in combination with fish oil, 9 (9.5%) in combination with a statin, and 1 on fibrate-niacin combination. Eight patients (8.4%) were placed on fish oil monotherapy, while one was left on statin monotherapy. At the last visit recorded (84 patients), 56 patients (66.7%) remained on fibrate therapy, with 31 patients (36.9%) on monotherapy, 11 (13.1%) in combination with a statin, 4 (4.8%) in combination with fish oil, and 1 in combination with niacin. Six patients (7.1%) were on statin monotherapy, 3 (3.6%) on fish oil monotherapy, and 2 (2.4%) on niacin monotherapy. Of the remaining 25 patients, 17 (20.2%) were on combination therapy without a fibrate, and 8 (9.5%) were on no treatment.

**Table 5:** Prevalence of Lipid-lowering Medications in Clinic HTG Patients

		Upon Arrival*	Clinic Treatment*	Latest Visit**
		n (%)	n (%)	n (%)
Monotherapy:	Fibrate	17 (17.9)	54 (56.8)	31 (36.9)
	Statin	16 (16.8)	1 (1.1)	6 (7.1)
	Fish Oil	1 (1.1)	8 (8.4)	3 (3.6)
	Niacin	1 (1.1)	0	2 (2.4)
	Resin	1 (1.1)	0	1 (1.2)
Combination:	Fibrate/Statin	4 (4.2)	9 (9.5)	11 (13.1)
	Fibrate/Niacin/Fish Oil	1 (1.1)	1 (1.1)	1 (1.2)
	Fibrate/Fish Oil	1 (1.1)	16 (16.8)	4 (4.8)
	Other	0	2 (2.2)	17 (20.2)

* N = 95, ** N = 84

### Laboratory and Anthropometric Values of Patients from Initial to Latest Visit (Table 6)

**Figure 1:**
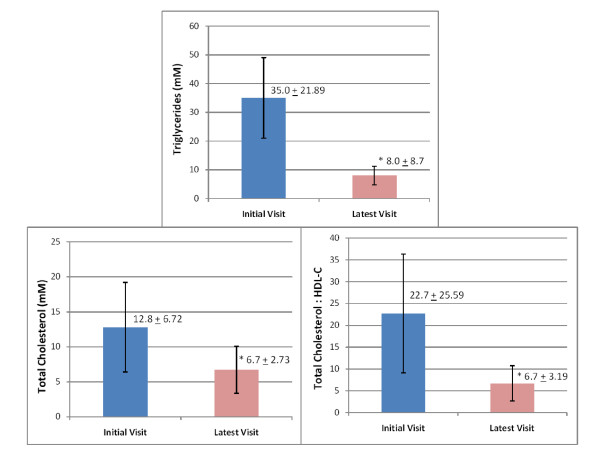
**Triglycerides, Total Cholesterol, and Total Cholesterol:HDL-C Ratio at Initial and Latest Clinic Visit in HTG Patients**. *, significant difference between means, *p *< 0.001 (two tail; pair student t-test).

Triglyceride levels decreased from a mean of 35.04 ± 21.89 mM (3104 ± 1939 mg/dl) at the initial visit to 8.07 ± 8.71 mM (715 ± 772 mg/dl) at the latest visit (Figure 1). Total cholesterol (TC) dropped from 12.8 ± 6.37 mM (495 ± 247 mg/dl) to 6.72 ± 2.73 mM (260 ± 106 mg/dl), and TC to high density lipoprotein cholesterol (HDL-C) ratio from 22.7 ± 25.59 to 6.70 ± 3.19 (Figure 1). No statistically significant changes were detected for HDL-C. Average BMI was unchanged from first to the latest visit.

**Table 6:** Laboratory Values and Anthropometry of HTG Patients from Initial to Latest Visit

		Initial Visit (mM)	Latest Visit (mM)	Paired t - test
Laboratory:	Triglycerides	35.04 ± 21.89	8.07 ± 8.7	p < 0.0001
	TC	12.8 ± 6.37	6.72 ± 2.73	p < 0.0001
	TC/HDL-C	22.7 ± 25.59	6.70 ± 3.19	p < 0.0001
	HDL-C	0.87 ± 1.05	1.05 ± 0.31	p = 0.224
	Systolic BP	126.91 ± 18.12	128.11 ± 19.96	p = 0.634
	Diastolic BP	80.46 ± 10.05	80.46 ± 10.05	p < 0.05
Anthropometric:	BMI	29.61 ± 5.17	29.55 ± 6.27	p = 0.917

TC = Total Cholesterol; HDL-C = High Density Lipoprotein Cholesterol; BP = Blood Pressure; BMI = Body Mass Index

## Discussion

### Clinical findings in patients with severe HTG

The incidence of clinical findings including pancreatitis and physical stigmata in patients with severe HTG have not previously been documented. Other studies have reported average acute levels of plasma TG in patients with TG-induced pancreatitis of 51.8 mM [8] and 50.5 mM [9], but did not define a lower limit of plasma TG at which HTG could be identified as the likely cause of the pancreatitis. A key finding of this study is that acute pancreatitis as a consequence of HTG occurs relatively infrequently, and rarely if ever unless TG levels are greater than 20 mM (1772 mg/dl). In this study a history of pancreatitis was present in 15.8% of individuals referred with TG > 20 mM, occurring in patients with a minimum non-acute TG level of 20.5 mM (1816 mg/dl) and a mean non-acute TG level of 38.1 mM (3376 mg/dl). Of 91 patients seen in our clinic with non-acute TG levels between 10-20 mM, none had a history of pancreatitis unless their TG were > 20 mM acutely. Eruptive xanthomas were found in only 8.5% of patients, at a minimum TG level of 20.5 mM (1816 mg/dl) and an average TG level of 67.8 mM (6007 mg/dl), even higher than that required to induce pancreatitis. However, the level of TG did not accurately predict whether patients would develop these signs. For instance, pancreatitis was observed in 2 patients with TG levels of 20-21 mM, and only in 1 out of 5 patients who had TG>70 mM. It is likely that factors other than the TG level may contribute to the development of pancreatitis and other physical characteristics observed in HTG patients. Further studies with a greater sample size and possibly knowledge of the underlying genetic traits are needed to better elucidate such relationships. Lipemia retinalis was an infrequent finding in our study. While it was uncertain whether this was assessed at the time of non-acute presentation in all patients, it appears that this finding is present mainly in very severe HTG and more likely to be seen in the acute versus non-acute HTG setting.

Chylomicronemia syndrome, defined as TG > 1000 mg/dl (11.3 mM) plus one of either eruptive xanthomas, lipemia retinalis, or abdominal pain/pancreatitis, had a previously quoted incidence rate of 1.7/10000 patients (< 0.02%) [10]. Recent increases in obesity and DM rates, however, have led to a potential increase in this incidence. In our high risk patient population, 23 patients (24.2%) met the criteria for this syndrome.

### Secondary contributors to severe HTG

In addition to inherited causes of HTG that were undoubtedly present in most if not all the patients in this cohort, the most common secondary factors predisposing to HTG were a high fat and carbohydrate diet, physical inactivity and obesity. In 53% of the cohort, and concomitant with these other factors in many cases, dysglycemia was present in the form of pre-diabetes or overt Type 2 diabetes mellitus. Two previous studies found similarly high levels of diabetes, 72% [8] and 43% [9], in acute HTG-induced pancreatitis. The negative impact of metabolic syndrome, dysglycemia and abdominal obesity on triglyceride levels is well documented [1,3,11]. Insulin resistance and diabetes are associated with an increase in plasma TG for multiple reasons, including reduced insulin-dependent inhibition of lipolysis in adipocytes, increased TG and VLDL production by the liver, and impaired insulin-dependent activation of lipoprotein lipase and hydrolysis of TG-rich lipoproteins [3,12].

Although known to contribute to HTG, the incidence of uncontrolled DM and hypothyroidism has not been well characterized in previous studies. The majority of patients (77%) with DM at first presentation to the clinic had poor glycemic control. Diagnosed hypothyroidism was present in 17.1% of patients, with a significant number of these (33.3%) having evidence of inadequate thyroid hormone replacement (TSH > 5 uIU/mL). The high incidence of such comorbidities highlights the importance of controlling these factors to prevent severe HTG. All told, 100% of our patients had at least one secondary factor contributing to their HTG. Although genetic testing is not currently available for familial combined hyperlipidemia and is not routinely available for many other inherited causes of HTG, our assumption is that in most if not all of our patients a combination of primary genetic plus secondary causes contributed to their severe HTG. In any patient with TG levels >3-4 mM, it should be assumed there is a likely underlying inherited cause of HTG present, aggravated further by one or more secondary factors.

### Lipid Lowering Therapy

At initial visit, almost as many patients were being treated by their referring physician with statin monotherapy as fibrate monotherapy, the recommended first line treatment for severe HTG. Lipid specialists, on the other hand, treated most patients with fibrate monotherapy. It has been well documented that fibrates are the most efficacious first line pharmacotherapy for HTG, and typically lower TG very rapidly and effectively [3,11,13]. Adjuncts to this include lifestyle modifications, such as following a low fat and low simple carbohydrate diet, avoiding alcohol, control of blood sugars, and fish oil Ω-3 fatty acid) supplementation [3,14]. Fish oil doses of at least 2 g in doses divided two or three times daily are needed to have any major TG-lowering effect, and doses of up to 12 g daily can be used. Niacin (nicotinic acid) is also a potent TG-lowering agent. It is not practical to use in the acute setting, however, where reducing the risk of pancreatitis is the main priority, given the time needed to titrate the niacin dose to therapeutic levels (≥ 1000 mg daily). In most cases, apart from homozygous lipoprotein lipase or apolipoprotein CII deficiency, use of a fibrate in combination with a low fat and carbohydrate diet and improvement in blood sugar control is highly efficacious in reducing severe HTG to more moderate levels, as seen in our cohort. While statins have some TG-lowering effect, ranging from 20 - 28% at higher doses of newer statins [15], they are not effective enough in this regard to remove the risk of pancreatitis in patients with severe HTG, and should not be used on their own as first line agents. The discrepancy between treatments initiated by referring physicians and lipid specialists needs to be addressed, perhaps by an education series focused on lipid treatment. By doing so, it is possible that more patients would be able to achieve marked TG reduction without lipid clinic referrals.

In patients with severe HTG and persistent elevation of apolipoprotein B100 or LDL-C following initiation of TG-lowering therapy, treatment with combined fibrate/statin may be indicated. Although the recent ACCORD Lipid trial did not find additional benefit of adding fenofibrate to a statin in terms of cardiovascular event rate in patients with type 2 diabetes [16], it may be necessary to continue fibrate therapy long term in severe HTG patients who also require a statin, in order to maintain TG levels out of the range of risk for pancreatitis. While we did not observe pancreatitis in anyone with TG levels < 20 mM, due to the volatile nature of TG elevation when secondary factors are not well controlled, we recommend attempting to lower TG levels to not more than a maximum of 6-8 mM in order to reduce the risk of TG rising into pancreatitis range acutely.

## Conclusions

This is the first cohort in which incidence rates of clinical findings in patients with severe HTG have been quantified. The classic findings of pancreatitis and eruptive xanthomas occurred in a relatively small percentage of patients, and not unless TG were > 20 mM (1772 mg/dl). Patients with extreme HTG have a combination of primary and secondary factors contributing to their HTG. Lifestyle changes (low fat and simple carbohydrate diet, increased exercise) and a reduction in comorbidities (uncontrolled DM and hypothyroidism) are critical aspects of managing HTG in conjunction with pharmacotherapy using fibrates and omega-3 fatty acid supplements. Fibrates remain the treatment of choice for severe HTG, with statins lacking sufficient TG-lowering effect to remove the risk of pancreatitis.

## Methods

A retrospective chart review was conducted in the Healthy Heart Program Lipid (now Prevention) Clinic at St. Paul's Hospital, Vancouver, Canada. Patients seen in the clinic between the years of 1986 and 2007 with plasma triglyceride levels greater than 20 mM upon referral were included in the study. Patients were identified either by individual chart review, or after 1999 by scan of an electronic clinic database. In total, 95 patients met this inclusion criterion; of those, 84 patients returned for at least one follow up visit, and many patients have been followed intermittently for several years. Data extracted from the chart from the patient's initial visit and most recent follow up visit for the current study included: demographics (age, gender, ethnic background); history of pancreatitis; presence of increased fasting glucose or diagnosis of diabetes at initial or follow up visit; other historical features including secondary factors potentially contributing to the presence of HTG (dietary carbohydrates and fats, level of physical activity, alcohol consumption, hypothyroidism, current smoking, and medications potentially raising TG including thiazide diuretics, β-blockers, oral estrogen, and anti-retroviral agents), history of cardiovascular disease, and family history of premature vascular disease or known dyslipidemia; physical findings including body mass index, dermatologic findings including eruptive or palmar xanthomas, eye findings including corneal arcus and lipemia retinalis, and abdominal findings including epigastric tenderness and hepatomegaly. Use of lipid-lowering therapies including fibrates (gemfibrozil or fenofibrate), statins (simvastatin, pravastatin, atorvastatin or rosuvastatin), niacin, omega-3 fatty acids (fish oils), and bile acid binding resins were recorded. Total cholesterol, triglyceride levels, HDL-cholesterol (HDL-C) and total cholesterol:HDL-C ratio were recorded for the initial and follow up visits. This study was approved by the Institutional Ethics Review Board for Human Studies, Providence Health Care Research Institute, Vancouver, BC, Canada.

### Statistical Analysis

The cohort's characteristics were expressed as mean and standard deviation for continuous variables. Frequency of findings was used for dichotomous traits. Means were compared using a paired Students t test, when normality was confirmed, two tailed - 95% CI with a *p *value < 0.05 considered statistically significant. Data analysis was performed using SPSS v12.0 (SPSS Inc., Chicago, IL).

## Competing interests

The authors declare that they have no competing interests.

## Authors' contributions

SS participated in the collection and analysis of data and writing of the manuscript. AA-S and CT participated in the data collection and analysis. JF participated in conception of the study, supervision, data analysis and manuscript editing. GF participated in conception and oversight of the study, supervision, data analysis and manuscript preparation. All authors read and approved the final version of the manuscript.
